# AQP7-Mediated Mitochondrial Redox Homeostasis in Vitrified Oocytes: A Genetic Mechanism of PI3K/AKT Signaling Regulation

**DOI:** 10.3390/genes16070730

**Published:** 2025-06-23

**Authors:** Yatian Qi, Wei Xia, Chenyu Tao, Xiaohuan Fang, Yang Yu, Jingwei Hu, Xiaofeng Tian, Tianmiao Qin, Congcong Yao, Wentao Zhang, Junjie Li

**Affiliations:** 1 College of Animal Science and Technology, Hebei Agricultural University, Baoding 071001, Chinafangxiaohuan94@163.com (X.F.); yyang980828@163.com (Y.Y.); hujingwei2024@163.com (J.H.); qintianmiao@163.com (T.Q.); 18733937125@163.com (W.Z.); 2 Hebei Technology Innovation Center of Cattle and Sheep Embryo, Baoding 071001, China; xiaweihawaii@163.com (W.X.);

**Keywords:** AQP7, vitrification, oocyte cryopreservation, PI3K/AKT signaling, mitochondria, genetic regulation

## Abstract

Background/Objectives: Cellular oxidative stress is crucial for GV stage oocyte vitrification quality. PI3K and the aquaporin family have been shown to facilitate various cellular processes related to redox homeostasis and energy balance; yet, the mechanisms underlying the involvement of aquaporin 7 (AQP7) in vitrified oocyte oxidative stress remain unclear. The purpose of the present investigation was to evaluate the role of AQP7 in vitrified oocytes and the mechanisms involved. Methods: AQP7 inhibitors were employed to investigate the effect of AQP7 on oxidative stress in mouse-vitrified oocytes, whereas PI3K activators were harnessed to ascertain whether AQP7 serves as a functional molecule involved in this process. Results: Our results indicate that AQP7 inhibition in vitrified oocytes results in a significant decrease in glutathione (GSH) levels associated with cellular oxidation and an elevation in H_2_O_2_ levels. This was accompanied by exacerbated mitochondrial dysfunction, weakened cytoskeletal proteins, accelerated early apoptosis. Consequently, both survival and maturation rates were markedly reduced. Interestingly, PI3K/AKT activation increased AQP7 expression, restored abnormal mitochondrial distribution, as well as calcium homeostasis, and rescued the oocyte survival/maturation rate. Conclusions: Our results provide new insights indicating that PI3K/AKT/AQP7 decreases oxidative stress by regulating mitochondrial morphology, function, and distribution, thereby rescuing oocyte maturation in vitrification.

## 1. Introduction

Cryopreservation of germinal vesicle (GV) stage oocytes provides a stable source of oocytes, enabling the establishment of oocyte banks and enhancing the utilization rate of oocytes. This process is of significant importance not only for clinical applications in human-assisted reproduction but also for the preservation of genetic resources in seasonally bred animals and endangered species [1,2]. Vitrification, which utilizes high concentrations of cryoprotectants to achieve rapid dehydration, effectively inhibits the formation of ice crystals. This method has been demonstrated to be a highly efficient ultra-rapid cooling technique, and its potential as a cryopreservation method was first shown before 1985 [3].

The deterioration of vitrified oocyte quality is significantly influenced by oxidative stress. During oogenesis and early embryonic development, mitochondria precisely regulate redox homeostasis, which profoundly affects the activity of biomolecules and signaling pathways [4,5].

Approximately 1% of molecular oxygen undergoes transformation into superoxide anions as a consequence of mitochondrial electron leakage during an electron transport chain reaction, promptly initiating the production of hydrogen peroxide (H_2_O_2_) [6,7]. H_2_O_2_ can then engage in a Fenton reaction, leading to the continuous generation of hydroxyl radicals. Importantly, hydrogen peroxide is a key reactive oxygen species (ROS) that fundamentally influences cellular survival, proliferation, and differentiation processes [8]. The augmentation of ROS levels as a consequence of vitrification serves as a prominent inducer of oxidative stress [9,10], and excess ROS induces cellular damage [11,12], such as a disruption of mitochondrial ultrastructure, distribution, and function [13,14].

Phosphoinositide-3 kinase (PI3K) plays a crucial role in oocyte vitrification. Specifically, the PI3K/Akt signaling pathway exhibits a strong correlation with oxidative stress and developmental potential in oocytes vitrification [15]. Upon activation, this pathway promotes oocyte maturation by alleviating oxidative stress, mitochondrial dysfunction, and cellular apoptosis [16,17]. Additionally, it has been reported that PI3K enhances the tolerance of vitrified oocytes by regulating the AQP7 channel [18]. This further underscored the multifaceted role of PI3K in supporting the success of oocyte vitrification.

AQP7, a member of the aquaglyceroporin subfamily within the aquaporin protein family, is characterized by its unique pore architecture comprising six transmembrane α-helices. Notably, two of these helices exhibit atypical half-transmembrane configurations, forming a central pore conduit. Beyond its canonical role in water transport, AQP7 has been identified as a facilitative transporter for hydrogen peroxide (H_2_O_2_) [19], a reactive oxygen species (ROS) that serves as a critical secondary messenger in cellular redox signaling pathways [20].

Previous studies have demonstrated that *AQP7* knockdown significantly reduces oocyte survival after vitrification [18]; yet, the molecular mechanisms underlying AQP7-mediated cryotolerance remain elusive. Given the established role of H_2_O_2_ in both oxidative stress responses and redox signaling, we hypothesize that AQP7 may improve tolerance to vitrified oocytes by ameliorating mitochondrial oxidative stress.

Based on these, to assess the role of AQP7 in vitrified GV oocytes and elucidate the mechanism by which PI3K enhances the tolerance of vitrified oocytes after thawing through AQP7, we employed immunohistochemical, immunofluorescence staining, and other molecular biology techniques. These approaches allowed us to assess the impact of PI3K/AKT/AQP7 on survival rates, maturation rates, oxidative stress and apoptosis of GV oocytes undergoing vitrification.

## 2. Materials and Methods

### 2.1. Animals and Shelter

Six-week-old female ICR mice were acquired from SPF Biotechnology Co., Ltd. (Beijing, China). The mice were housed in well-ventilated environments under controlled temperatures and humidity, with free access to water/food. They were maintained on a standard photoperiod of 12 h of light and 12 h of darkness. The animal maintenance and experimental operations in this experiment were conducted according to the Guidelines for the Management and Use of Laboratory Animals at Hebei Agricultural University.

### 2.2. Chemicals and Antibodies

All medications and chemicals were purchased from Sigma (St. Louis, MO, USA) unless stated otherwise. The anti-AKT antibody, anti-p-AKT antibody, and anti-p-PI3K antibody were from Cell Signaling Technology (Cell Signaling, Danvers, MA, USA). The anti-AQP7 antibody and anti-PI3K antibody were from Santa Cruz (Dallas, TX, USA). The Fluorescein (FITC)-conjugated affinity goat anti-rabbit IgG (H + L) secondary antibody and Fluorescein (FITC)-conjugated affinity goat anti-mouse IgG (H + L) secondary antibody were from Proteintech (Beijing, China).

### 2.3. Oocyte Collection

After a two-week acclimation period, the mice were injected intraperitoneally with 10 IU Pregnant Mare Serum Gonadotropin (PMSG, Ningbo No. 2 Hormone Factory, Ningbo, China). Following a period of 44 to 48 h, the mice were euthanized by cervical dislocation. The excised ovaries were promptly transferred to an M2 solution supplemented with 2.5 μM milrinone at 37 °C. The ovarian follicle was punctured with a 1 mL syringe to collect the GV-stage cumulus–oocyte complexes (COCs). COCs from 4 to 6 mice per experimental group were pooled to minimize inter-individual variability; the COCs pooled from 4 to 6 mice per experimental group were combined. Only oocytes with 2–3 layers of cumulus cells were cryopreserved for vitrification.

### 2.4. Vitrification and Thawing of Oocytes

The vitrification and thawing solutions were bought from AibeiBio (Nanjing, China). Z433927330 (Selleck, Shanghai, China), an AQP7 inhibitor [20,21], was initially dissolved in DMSO to 10 mM and then added exclusively to the vitrification and warming solutions at different concentrations (0.5, 1, 5, 10 μM). No addition was made to the other solutions in the protocol. 740Y-P (Selleck, Shanghai, China), a PI3K activator, was first dissolved to 10 mM with DMSO and then specifically diluted in the vitrification/warming solutions (5 μM) without addition to the other solutions.

For vitrification, GV oocytes were equilibrated in ES solution for 5 min, then exposed to VS solution for 1 min at room temperature. Within 30 s, the COCs were precisely positioned onto the leaf of Cryotop (Beijing, China), and kept in liquid nitrogen (LN2) for at least 1 week.

For thawing, the leaf of the Cryotop was quickly immersed in the first thawing solution for 1 min. Then, the COCs were soaked in each of the other three thawing solutions for 3 min, respectively. After thawing, the COCs in the GV stage underwent an additional recovery period of 1 h before proceeding to the subsequent experiment.

### 2.5. Oocyte in Vitro Maturation (IVM)

An M16 medium was used to culture the COCs at 37 °C with 5% CO_2_. After 14 h of IVM, the COCs were treated with 0.1% hyaluronidase (Sigma, St. Louis, Missouri, USA) for 1 min, and first polar body extrusion (PBE) rates were recorded.

### 2.6. Immunohistochemical Staining (IHC) and Immunofluorescence (IF) Staining

To validate the localization of AQP7 in the oocytes residing within ovarian tissue, the ovary or oocytes were subjected to fixation with 4% polyformaldehyde (PFA) for 40 min, followed by a 1 h permeabilization process using 0.5% Triton X-100. After 1 h of blocking with 3% BSA (Zhejiang Tianhang Biotechnology Co., Ltd., Huzhou, China), they were further treated with primary antibodies overnight (anti-AQP7, 1:500; anti-PI3K, 1:100; anti-AKT, 1:100; anti-p-PI3K, 1:100; anti-p-AKT, 1:100) and the appropriate secondary antibodies for 1 h. Lastly, the samples were stained with 4′,6-diamidino-2-phenylindole (DAPI, Beyotime, Shanghai, China) for 3 min and images are taken with a fluorescence inverted microscope.

To stain F-actin, same as with the fixation and permeabilization steps mentioned above, the oocytes were next placed in phalloidin (green fluorescence), diluted 200-fold in accordance with the directions provided by the F-actin staining kit (Wuhan Albertin Technology Co., Ltd., Wuhan, China), stained for 30 min, and images were taken with a fluorescence inverted microscope.

### 2.7. Transmission Electron Microscopy (TEM)

The oocytes were fixed in 2.5% glutaraldehyde at 4 °C overnight, encapsulated in 1% agarose, and further fixed with 1% osmium tetroxide for 2 h. The sample underwent a series of dehydration treatments using ethanol and acetone, followed by infiltration and embedding in EMBed-812 resin (SPI, South Padre Island, TX, USA). Using an ultramicrotome (Leica, Wetzlar, Germany), the resin block is cut into ultrathin sections (60–80 nm) and fixed on a copper grid. After staining with uranyl acetate and lead citrate-enhanced contrast, the samples were examined using a transmission electron microscope (HT7700, Hitachi High-Tech Corporation, Tokyo, Japan).

### 2.8. Analysis of H_2_O_2_ and GSH Levels

To measure the H_2_O_2_ levels in oocytes, the methodology outlined by Khatun et al. [22] was employed. In short, the GV oocytes were put in 10 μM 2′,7′-dichlorofluorescein diacetates (2′,7′-DCFH-DA, Eugene, OR, USA) for 20 min. After incubation, the oocytes were washed and imaged using a fluorescence microscope (ECLIPSE Ti-s, Nikon, Tokyo, Japan). The intensity was then quantified utilizing ImageJ software (version 1.51j8; NIH, Bethesda, MD, USA).

To ascertain the GSH concentrations in the oocytes, a 20 min incubation period with a 10 μM solution of 4-chloromethyl-6,8-difluoro-7-hydroxycoumarin (Cell Tracker Blue, CMF2HC; Eugene, OR, USA) was conducted. Following the same protocol as above, the oocytes were washed, photographed, and subsequently analyzed for the intensity of their fluorescence emissions.

### 2.9. Detection of the Mitochondrial Distribution

The oocytes were incubated in 100 nmol/L Mito-Tracker Red CMXRos (Beyotime, China) for 20 min and then washed. A fluorescence microscope was set to excite red fluorescence at a wavelength of 579 nm for imaging. Subsequently, ImageJ software was used to quantitatively assess the intensity of this fluorescence.

### 2.10. Determination of MMP

JC-1 assay kits (Beyotime, Shanghai, China) were utilized to assess mitochondrial membrane potential (MMP). In the assay, the oocytes were incubated in a solution containing 10 mmol/L of JC-1 at 37 °C in an environment of 5% CO_2_ for a period of 20 min. Following this incubation, the oocytes were thoroughly washed with an M2 medium to eliminate unbound dye and then examined under a fluorescence microscope. The MMP was quantitatively determined by calculating the ratio of red fluorescence intensity to the green fluorescence intensity.

### 2.11. Determination of the Ca^2+^ Levels

The Fluo-4 AM kit (Invitrogen, Carlsbad, CA, USA) was used to determine the levels of Ca^2+^ in the oocytes. Briefly, the oocytes were exposed to a Fluo-4 AM solution for 30 min at a temperature of 37 °C. Following this, the oocytes underwent three washes with PBS and were subsequently imaged under a fluorescence microscope. The ImageJ software was utilized to evaluate the mean fluorescence intensity displayed by the oocytes.

### 2.12. Annexin V Staining

To evaluate the apoptotic status of oocytes, we employed an Annexin V staining kit sourced from Vazyme (Nanjing, China), adhering strictly to the guidelines. In summary, the oocytes were subjected to staining for 10 min at 37 °C using a solution comprising 100 μL of a binding buffer and 5 μL of Annexin V-FITC. Following staining, the fluorescent signals were meticulously analyzed under a fluorescence microscope, allowing for the assessment of the apoptotic state of the oocytes based on Annexin V binding.

### 2.13. Real-Time Quantitative PCR (qRT-PCR)

Following the guidelines provided by the extraction kit (Qiagen, Hilden, Germany), total RNA was extracted from 100 oocytes per group and stored at −80 °C for later use. The NanoDrop 2000 (Thermo, Waltham, MA, USA) was used to measure both the optical density ratio between 260 nm and 280 nm (1.9–2.1) and the total RNA concentration. A reverse transcription kit (TaKaRa, Tokyo, Japan) was utilized to converted RNA into cDNA.

qRT-PCR was performed using SYBR fluorescent dye (Biotium, Bay Area, CA, USA) on a QuantStudio 6 Flex Real-Time PCR System (Applied Biosystems, Foster City, CA, USA). The PCR protocol included an initial denaturation at 95 °C for 2 min, followed by 40 cycles of denaturation at 95 °C for 5 s and annealing/extension at 60 °C for 30 s. The forward primer of the *Aqp7* gene were designed based on NCBI reference sequences: TAGGCCGAATGACCTGGAA, and the reverse primer were designed: GAAGATAGGTGGCAAAAATGT. The *Gapdh* gene served as the reference gene (F: ACACTGAGGACCAGGTTGTCTC. R: TACTCCTTGGAGGCCATGTAG), and was evaluated by employing the comparative Ct (2^−ΔΔCt^) method.

### 2.14. Experimental Design

This study mainly consists of Exp. 1 and 2.

In Exp. 1, fresh GV COCs were arbitrarily assigned to three groups: a control group (Fresh group), a vitrification group (Vit group), and a Z433927330-treated vitrification group (Vit + Z433 group), where Z433927330 was incorporated into all vitrification and warming solutions. After thawing, measurements were taken for changes in AQP7 abundance, survival rate, maturation rate, cytoskeletal organization, early apoptosis rate, redox status, mitochondrial structure, function, and distribution.

In Exp. 2, fresh GV COCs were randomized into three groups: a Vit group, a Vit + Z433 group, and a Vit + Z433 + 740Y-P group. The Vit + Z433 + 740Y-P group involved the concurrent inclusion of 0.5 μM of Z433927330 and 5 μM of 740Y-P in all vitrification/thawing solutions. The changes in AQP7, PI3K, AKT, p-PI3K, and p-AKT abundance levels, cell survival and maturation rates, mitochondrial distribution, and Ca^2+^ levels were detected after thawing.

### 2.15. Statistical Analysis

All experiments are performed at least three times for repetition. Statistical analysis was performed using SPSS 21.0 software (IBM Corporation, Armonk, NY, USA) with one-way ANOVA, and the results were presented as mean  ±  standard error of the mean (SEM) (* *p* < 0.05, ** *p* < 0.01 for significant differences).

## 3. Results

### 3.1. Abundance of Aquaporin 7 in Mouse Ovaries

**Figure 1:**
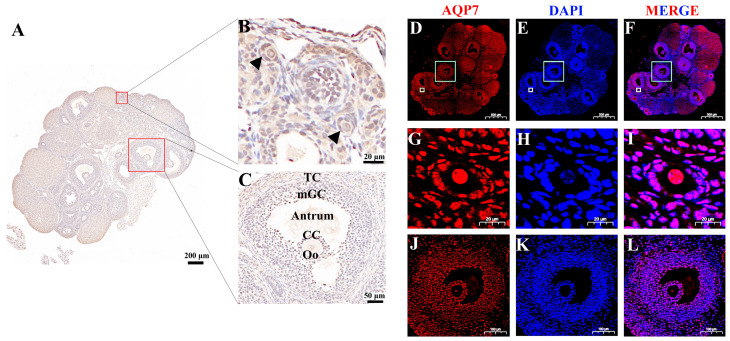
AQP7 abundance in mouse ovary and oocyte. Immunolocalization of AQP7 in mouse ovaries and oocytes. (**A**) Immunohistochemical localization of AQP7 in mouse ovaries, scale bar, 200 μm. (**B**) AQP7 immunolocalization in preantral follicles. Black arrows indicate positive signals; Scale bar, 20 μm. (**C**) AQP7 distribution in antral follicles, scale bar, 50 μm. Abbreviations: Oo, oocyte; CC, cumulus cells; Antrum, follicular antrum; mGC, mural granulosa cell; TC, theca cell. (**D**–**F**) Immunofluorescence co-staining of AQP7 (red) and nuclei (blue) in ovarian sections. Scale bar, 500 μm, Yellow and green boxes highlight preantral and antral follicles, respectively. (**G**–**I**) AQP7 and nucleus in preantral follicle, scale bar, 20 μm. (**J**–**L**) AQP7 and nucleus in antral follicle, scale bar, 100 μm.

The abundance of AQP7 in mice ovaries was assessed by immunohistochemistry and immunofluorescence. The results demonstrated widespread AQP7 expression throughout the ovarian tissue. Notably, AQP7 was prominently expressed in oocytes as well as granulosa cells, encompassing both antral and preantral follicles, indicating its widespread distribution and a potential functional significance in these critical ovarian compartments (Figure 1A–L).

### 3.2. Effect of AQP7 on Oocyte Viability After Vitrification

**Figure 2:**
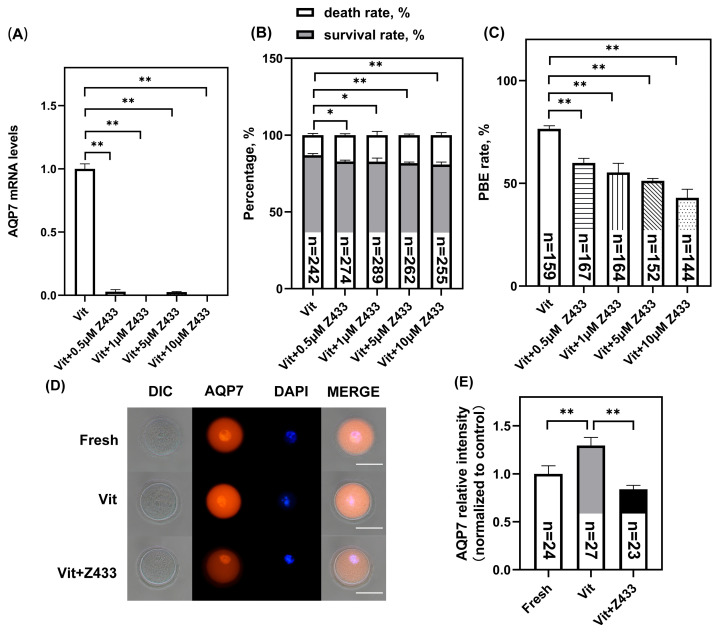
Effect of AQP7 on oocyte viability after vitrification. (**A**) AQP7 mRNA levels, (**B**) survival rates, and (**C**) maturation rates of GV-stage vitrified oocytes after treatment with 0, 0.5, 1, 5, and 10 μM concentrations of Z433927330. (**D**) Representative images of AQP7 fluorescence intensity in GV oocytes. Scale bar, 100 μm. (**E**) Fluorescence intensity of AQP7 signals, where “*n*” denotes the cell number. * *p* < 0.05, ** *p* < 0.01.

The oocytes were treated respectively with different concentrations of AQP7 inhibitors Z433927330 (0.5 μM, 1 μM, 5 μM, and 10 μM) during vitrification and thawing, since vitrification increases AQP7 abundance [23]. After thawing and recovery, the *AQP7* mRNA levels in Z433927330-treated groups showed significant concentration-dependent reductions compared to the Vit group (*p* < 0.01) (Figure 2A). Concurrently, both cell survival rates (*p* < 0.05) (Figure 2B) and maturation rates (*p* < 0.01) (Figure 2C) were significantly decreased. Since 0.5 μM Z433927330 was sufficient to significantly inhibit the AQP7 level in vitrified oocytes of the GV stage (Figure 2D, E), this concentration was selected for vitrification in later experiments.

### 3.3. Effect of AQP7 on Vitrified Oocyte Meiosis and Apoptosis

**Figure 3:**
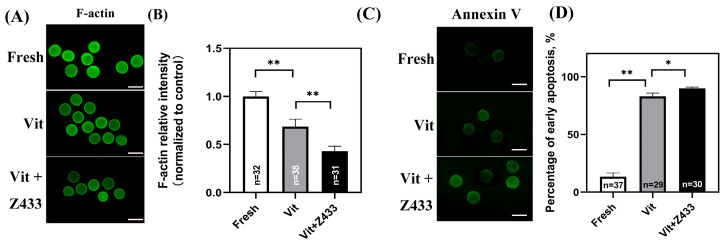
Effect of AQP7 on cytoskeleton and apoptosis. (**A**) F-actin staining in GV oocytes. Scale bar, 100 μm. (**B**) Quantification of F-actin fluorescence intensity. (**C**) Representative images of early apoptotic indicator Annexin V. Scale bar, 100 μm. (**D**) The rate of early apoptosis. “*n*” denotes the cell number utilized in the experiment. * *p* < 0.05, ** *p* < 0.01.

Actin is an essential constituent of the cytoskeleton and holds a crucial function during the meiosis process of oocytes in the GV stage. As shown, vitrification significantly reduced F-actin levels compared to the fresh group, which was further exacerbated by a 0.5 μM AQP7 inhibitor (*p* < 0.01) (Figure 3A,B). Annexin V is a crucial marker for evaluating oocyte quality and developmental potential, as it specifically binds to phosphatidylserine exposed on the surface of early apoptotic cells. Subsequently, the early apoptotic status of oocytes is assessed by using Annexin V probes. As shown in Figure 3, vitrification significantly increased the early apoptosis rate (*p* < 0.05). Compared with the vitrified group, additional AQP7 inhibition further significantly increased the early apoptosis rates (*p* < 0.05) (Figure 3C,D).

### 3.4. The Reduction in AQP7 Levels Affects Maturation Through Oxidative Stress Resulting from Damaged Mitochondria

**Figure 4:**
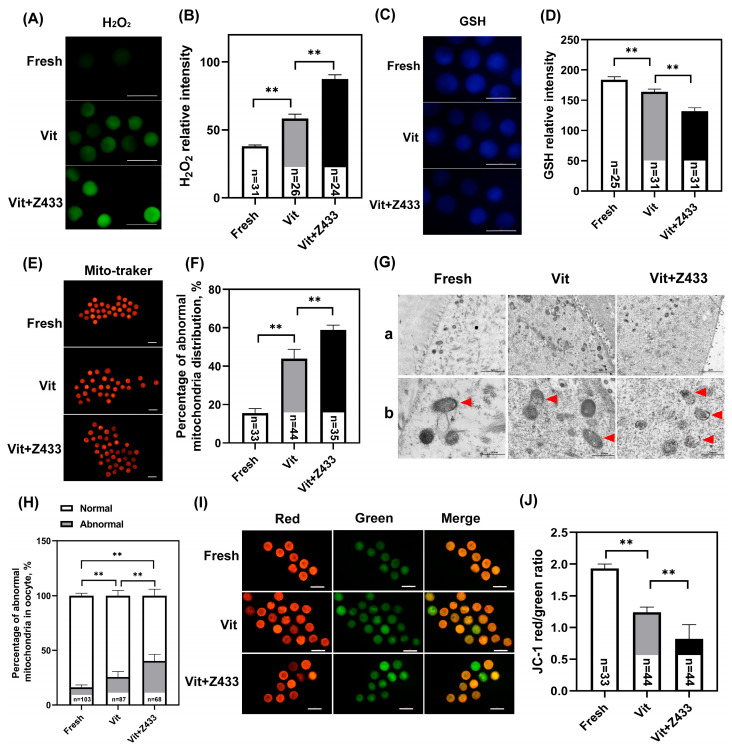
The reduction in AQP7 levels affects maturation due to oxidative stress caused by damaged mitochondria. (**A**) Levels of H_2_O_2_ in oocytes in different groups. Scale bar, 100 μm. (**B**) The fluorescence intensity of H_2_O_2_ signals. (**C**) Levels of GSH in oocytes in different groups. Scale bar, 100 μm. (**D**) The fluorescence intensity of GSH signals. (**E**) Mitochondrial distribution of oocytes. Scale bar, 100 μm. (**F**) Rate of abnormal mitochondrial distribution. (**G**) TEM images of vitrified GV-stage oocytes showing mitochondrial morphology (red arrows indicate mitochondria). (**a**) Magnification 1500×, scale bar, 2 μm; (**b**) Magnification 4000×, scale bar, 500 nm. (**H**) Rate of normal and abnormal mitochondrial distribution. (**I**) MMP was assessed in oocytes. Scale bar, 100 μm. (**J**) A quantitative analysis of MMP levels in different groups. ** *p* < 0.01.

The maintenance of cell viability relies on the redox state of oocytes, which was assessed by measuring the levels of H_2_O_2_ and GSH using 2′, 7′-DCFHDA and cell tracking blue, respectively. The decrease in AQP7 level exacerbated the oxidative stress, which was evident from the increased H_2_O_2_ levels (*p* < 0.01) (Figure 4A,B), and the decreased GSH levels (*p* < 0.01) (Figure 4C,D).

The structural integrity and functional stability of mitochondria are essential to alleviate oxidative stress in oocytes. Therefore, we first examined the mitochondrial distribution in oocytes. AQP7 inhibition caused more pronounced mitochondrial clustering compared to vitrified oocytes. (*p* < 0.01) (Figure 4E,F). TEM observations revealed ultrastructure changes in these mitochondria. We observed that the mitochondria in the GV oocytes in the fresh group were round or oval in shape, and the mitochondrial membrane was smooth and intact. In contrast, the mitochondria in the vitrified oocytes exhibited structural damage, including membrane disruption, with only a minority maintaining intact morphology. Notably, in the vitrified group treated with an AQP7 inhibitor, severe damage to the mitochondrial crest and mitochondrial membrane was observed (Figure 4G). In other words, the rate of mitochondrial abnormalities in Vit group was significantly increased (*p* < 0.01), while reduced AQP7 levels exacerbated mitochondrial structural damage (Figure 4H). Subsequently, the distribution of MMP was measured, revealing a decrease in the oocyte MMP in the Vit + Z433 group compared to the Vit group (*p* < 0.01) (Figure 4I,J).

### 3.5. PI3K/AKT/AQP7 Pathway Alleviate the Effect of GV Oocyte Vitrification

**Figure 5:**
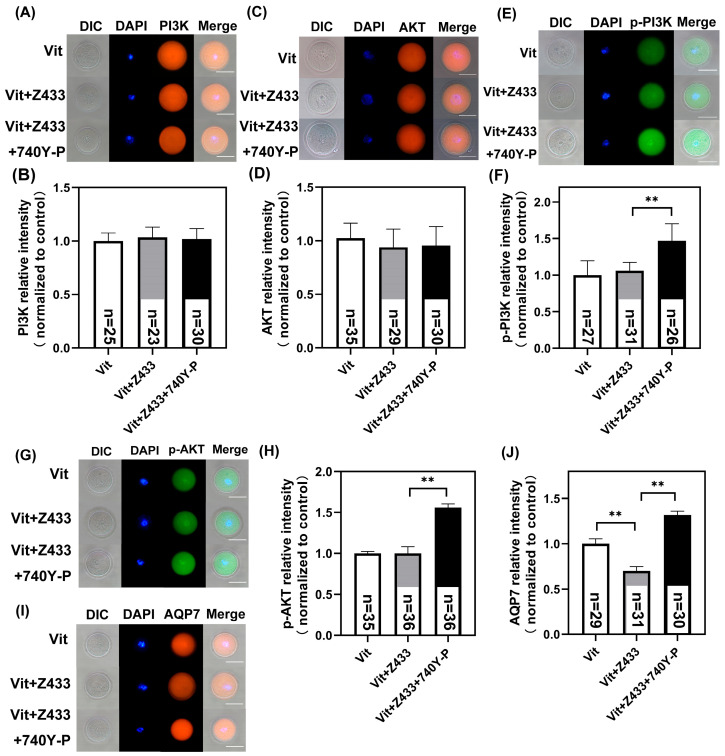
The PI3K/AKT pathway regulates AQP7 in vitrified oocytes. (**A**) PI3K, (**C**) AKT, (**E**) p-PI3K, (**G**) p-AKT, and (**I**) AQP7 staining of GV oocytes. Scale bar, 100 μm. Relative fluorescence intensities of (**B**) PI3K, (**D**) AKT, (**F**) p-PI3K, (**H**) p-AKT, and (**J**) AQP7 signals were detected. “*n*” represents the cell number. ** *p*  <  0.01.

Previous research indicates that insulin and leptin modulate AQP7 abundance via the PI3K pathway, and inhibiting this pathway reduces their impact on AQP7 abundance [24]. Therefore, we hypothesize that the PI3K/AKT pathway may mediate the regulation of AQP7 in oocytes to mitigate the effects of vitrification. To test this hypothesis, based on previous studies, we chose 5 μM 740Y-P to activate PI3K signaling [25]. As shown, treatment with 5 μM 740Y-P showed no difference in the total PI3K and AKT fluorescence signals among groups (*p* > 0.05) (Figure 5A–D). However, Vit + Z433 + 740Y-P treatment significantly increased p-PI3K and p-AKT fluorescence intensities compared to the Vit group and Vit + Z433 groups (*p* < 0.01) (Figure 5E–H), demonstrating that 5 μM 740Y-P successfully activates PI3K/AKT signaling in vitrified oocytes. Interestingly, compared to the Vit + Z433 group, the Vit + Z433 + 740Y-P group exhibited a notable elevation in AQP7 levels, demonstrating that the PI3K/AKT pathway regulates AQP7 abundance (*p* < 0.01) (Figure 5I,J).

### 3.6. Activated PI3K/AKT Restores Calcium Homeostasis, Mitochondrial Distribution, and Maturation Failure by Raising AQP7 Levels

**Figure 6:**
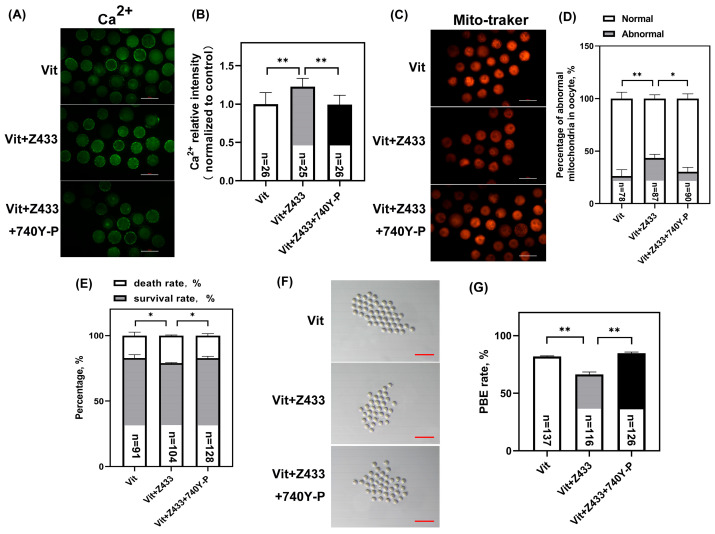
Activated PI3K/AKT restores calcium homeostasis, as well as mitochondrial distribution, and rescues maturation failure in AQP7-deficient oocytes. (**A**) Ca^2+^ levels of oocytes. Scale bar, 100 μm. (**B**) Quantification of Ca^2+^ fluorescence intensity. (**C**) Mitochondrial distribution of oocytes, scale bar, 100 μm. (**D**) Rate of abnormal mitochondrial distribution. “*n*” represents the cell number. (**E**) Rate of GV oocyte recovery after thawing. (**F**) After in vitro maturation of GV oocytes, PBE rates were quantified. Scale bar, 300 μm. (**G**) The PBE rates of oocytes. * *p* < 0.05, ** *p*  <  0.01.

The regulation of calcium homeostasis in oocytes is pivotal for ensuring their subsequent developmental competence. We detected calcium in the vitrified GV oocytes. Notably, 740Y-P attenuated the increase in Ca^2+^ caused by the group Vit + Z433 (*p* < 0.01) (Figure 6A,B). Mitochondrial distribution is critical for oocyte quality. As shown, the rate of mitochondrial abnormalities was significantly higher in the Vit + Z433 + 740Y-P group than in the Vit + Z433 group (*p* < 0.05) (Figure 6C,D).

As shown, oocyte recovery rates were improved in the Vit + Z433 + 740Y-P group compared to the Vit + Z433 group (*p* < 0.05) (Figure 6E), and the maturation rate was significantly increased (*p* < 0.01) (Figure 6F,G), suggesting that the tolerance of vitrified oocytes was improved by the activation of AQP7 through the PI3K/AKT pathway.

## 4. Discussion

AQP7 is expressed in various organs, tissues, and cells, including lipids, female reproductive systems, and skeletal muscles [26,27]. It facilitates the transport of small molecules such as glycerol, ammonia, urea, and arsenite [28]. According to this study, AQP7 was expressed in ovaries, especially in antral and preantral follicles. Analogously, the AQP7 protein is expressed in oocytes and granulosa cells at different stages in human and sheep ovaries [26,29]. Interestingly, we observed that the abundance of AQP7 was significantly higher in the granulosa cells adjacent to the follicular fluid compared to distal granulosa cells of the same type. This phenomenon is probably in accordance with the established function of AQP7 as a cell membrane channel protein [30].

To explore the effects of AQP7 in vitrified GV oocytes, we employed the specific AQP7 inhibitor Z433927330 to reduce AQP7 abundance. Consequently, both the survival rate and the PBE rate showed a significant decrease. While after the AQP7 levels were restored, oocyte survival and PBE rates were improved, which suggests that AQP7 positively influences the vitrification process of GV oocytes. In mouse oocytes, during the GV stage, the nucleus is enveloped by a layer of actin, and the polymerization of actin filaments has a beneficial effect on germinal vesicle breakdown (GVBD) [31]. Actin contributes to the maintenance of oocyte euploidy by inhibiting premature chromatid separation and facilitating the rearrangement of misaligned chromatids [32,33,34,35], profoundly affecting subsequent development [36,37,38]. The reduced oocyte maturation rate may be attributed to decreased F-actin levels. We noticed that after AQP7 was inhibited, the abundance of the cytoskeletal protein F-actin decreased, and the rate of early apoptosis increased. As a type of cytoskeletal protein, actin has also been implicated in the transport of numerous intracellular proteins [39]. Previous findings have demonstrated that protein trafficking engages actin and actin-based cytoskeletal complexes [40]. The translocation of AQP7 from the cytoplasm to the plasma membrane constitutes a critical step in oocyte survival, potentially requiring functional F-actin filaments to facilitate its membrane insertion and stabilization [18]. In simple terms, AQP7 and the cytoskeletal protein F-actin interact with each other, jointly influencing oocyte maturation.

Disruptions in cytoskeletal protein networks and the initiation of apoptotic cascades arise from oxidative stress-induced cellular dysfunction. This dysfunction stems from redox imbalance, which simultaneously compromises actin cytoskeleton integrity and activates pro-apoptotic signaling pathways [41]. The results of this study showed that after AQP7 was inhibited, the level of H_2_O_2_ increased, the level of GSH decreased, and mitochondrial morphology and function were seriously damaged. Our findings also indicate that upon restoration of AQP7 levels, the mitochondria was more evenly distributed. GSH is regarded as an important biochemical indicator for assessing their quality, and it is essential for maintaining the balance of redox reactions, thereby helping to protect oocytes from potential damage caused by oxidative stress [42]. An excessive accumulation of H_2_O_2_ induces severe oxidative stress, which is detrimental to the normal physiological operations of cells. Vitrification notably elevates the levels of ROS within the oocytes [43], which not only severely impairs the endogenous antioxidant defense system [44], but also consequently reduces oocyte survival rates and developmental competence [45]. The free radical, generated through the Fenton reaction of H_2_O_2_, exhibits exceptional reactivity—rapidly oxidizing amino acids and inducing irreversible protein conformational disruptions [8]. Hence, the transit of H_2_O_2_ across the plasma membrane into the extracellular milieu is also recognized as a possible way to remove oxidation [45]. AQPs are known to mediate transmembrane H_2_O_2_ diffusion and regulate downstream intracellular signaling pathways in mammalian cells, indicating their critical physiological roles [46,47,48]. AQPs facilitate the diffusion of H_2_O_2_ across membranes, affecting cell signaling and mitigating oxidative stress [49]. Among them, AQP7 has been identified to facilitate the transport of H_2_O_2_ [50]. In this study, AQP7 upregulation enhances mitochondrial structure and functional integrity in oocytes, serving as a protective mechanism for adaptation to vitrification.

Low temperature-induced endoplasmic reticulum (ER) stress elevates hydrogen peroxide (H_2_O_2_) levels [51], which severely disrupts intracellular calcium homeostasis and redox balance, ultimately compromising oocyte quality. As the primary cellular calcium reservoir, ER stress triggers the release of Ca^2+^ from the ER lumen into the cytoplasm, ultimately resulting in mitochondrial dysfunction [52]. This cascade—comprising ER stress, mitochondrial dysfunction, and elevated H_2_O_2_ levels—synergistically promotes cell apoptosis, impacting the developmental potential of oocytes. Our study revealed that AQP7 restoration significantly reduces abnormally high ER calcium levels, highlighting its critical role in maintaining ER calcium homeostasis.

The PI3K/AKT pathway, which is firmly linked to cellular oxidation and apoptosis, serves as a vital signaling cascade essential for various physiological functions, including cell development and survival, in response to extracellular signals [53,54].

The phosphorylation of PI3K activates its downstream protein AKT, leading to the formation of phosphorylated AKT. This activated form of AKT then regulates physiological functions such as cell proliferation, migration, and apoptosis by inhibiting a variety of the downstream effectors associated with programmed cell death [55,56,57,58]. Our results showed that AQP7 levels were still restored after the inhibition of AQP7 while activating the PI3K/AKT pathway, suggesting that the effect of AQP7 on oocytes was mediated by PI3K/AKT. Similarly, by activating the PI3K/AKT pathway to upregulate the abundance of AQP7, it promotes the proliferation and differentiation of mouse endometrial stromal cells during decidualization [59]. AQP7 abundance has been shown to be positively correlated with AKT phosphorylation [60]. These findings carry profound implications for elucidating the relationship between the PI3K/AKT pathway and AQP7, particularly under conditions of cellular oxidative stress and developmental processes.

## 5. Conclusions

Vitrification leads to an elevation of oxidative stress in GV oocytes.

**Figure 7:**
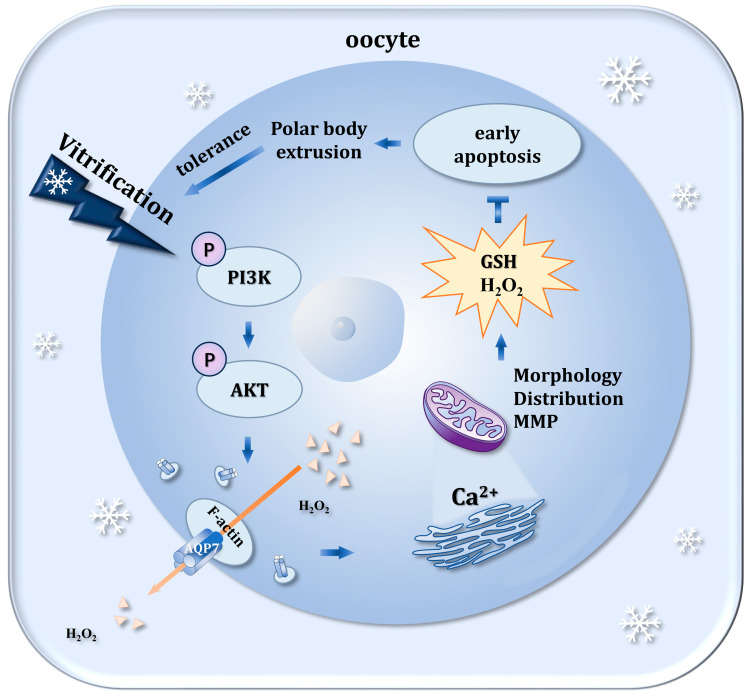
The PI3K/AKT/AQP7 pathway regulates antioxidant activity in GV-stage oocyte vitrification. Reduced AQP7 levels compromised mitochondrial function, disturbed cellular redox homeostasis, and disrupted spindle assembly, ultimately triggering early apoptosis and impairing polar body extrusion. In vitrified oocytes, PI3K/AKT-mediated phosphorylation upregulated AQP7 protein expression, which maintained intracellular calcium homeostasis and proper mitochondrial distribution, thereby enhancing cryotolerance during vitrification.

Our results indicated that the PI3K/AKT/AQP7 pathway modulates mitochondrial function in vitrified GV oocytes by regulating H_2_O_2_ levels, which subsequently reduces oxidative stress, maintains intracellular calcium homeostasis, and intacts cytoskeletal integrity. This pathway ultimately reduces apoptosis and promotes oocyte recovery and maturation. In addition, these findings extend our understanding of the role of AQP7 in antioxidant defense during GV-stage oocyte vitrification (Figure 7).

## Data Availability

The original contributions presented in this study are included in the article. Further inquiries can be directed to the corresponding authors.
